# Research on Coated Fabrics Dedicated to the Development of Artificial Transverse Muscle

**DOI:** 10.3390/ma18184225

**Published:** 2025-09-09

**Authors:** Łukasz Frącczak, Małgorzata Matusiak

**Affiliations:** 1 Lodz University of Technology, Faculty of Mechanical Engineering, Institute of Machine Tools and Production Engineering, 90-924 Lodz, Poland; 2 Lodz University of Technology, Faculty of Material Technologies and Textile Design, Institute of Architecture of Textiles, 90-924 Lodz, Poland; malgorzata.matusiak@p.lodz.pl

**Keywords:** woven fabric, polyester, silicone, coating, surface, roughness, artificial muscle

## Abstract

The aim of the present work was to select the thickness of a silicone layer coating a PES woven fabric designed for application in the artificial transverse muscles as a component of the medical robot. The artificial muscle in the form of U-shaped tube is subjected to repeated bending. Due to this fact, an important task was to ensure fatigue resistance to the bending of the muscle component. The fatigue bending was performed using the STM 601/12 device manufactured by SATRA Technology, Northamptonshire, UK. The surface geometry of the fabric before and after coating, as well as after 4000, 10,000 and 20,000 cycles of bending, was assessed using the MicroSpy^®^ Profile profilometer manufactured by the FRT, the art of metrology, Bergisch Gladbach, Germany. Additionally, the microscopic observations of fabric surface were performed after the abovementioned cycles of fatigue bending. The results obtained showed that in order to ensure the required functionality of the coated fabric, the 0.2 mm silicone layer is better than the 0.1 mm silicone layer.

## 1. Introduction

Medical engineering is an interdisciplinary field that combines knowledge from technical, biological, and medical sciences. Medical engineering deals with the design, development, and implementation of new technologies and solutions in medicine, such as medical equipment, software, biomaterials, and diagnostic methods. Medical engineering is also defined as an application of engineering principles and techniques to solve problems in medicine. This is evident across the healthcare spectrum from diagnosis and analysis to treatment and rehabilitation through the proliferation of implantable medical devices, such as pacemakers and artificial joints, to biotechnology. Included in the area of medical engineering are medical textiles. They are defined as specialized textile materials designed for use in healthcare and medical applications, ranging from basic wound care to complex implants and regenerative medicine.

Within medical textiles, two types are distinguished [1,2]:

- Non-implantable textiles (out-of-body), used for external applications like bandages, hospital garments, dressings, sutures, orthoses, and surgical gowns;
- Implantable textiles (in-body) (mostly tissue engineering), incorporated into devices like sutures, stents, and meshes, and requiring high biocompatibility and specific mechanical properties.

An application of textile materials for medical purposes very often requires a modification of textile surface [3]. A surface treatment of textile materials gives the desirable properties for particular medical application. In fabric surface treatment, different advanced processes are used, including printing, dyeing, and other treatments—chemical or physical—which allow the textile materials to be given unique functions. For instance, protection against UV radiation, wettability, and others. The coating of textile materials is one of processes applied in preparation of textile material for medical application. The original methods of coating were based on various impregnating techniques. However, when the coating was required only on one side of the material, the immersion of the fabric in the coating liquor was not possible. This stimulated the development of other coating techniques. Depending on the specific purpose, different coating processes can be used. We should mention here the following methods: knife coating, gravure coating, lick roll, rotary screen coating, hot-melt coating, and transfer coating [4].

Investigations showed that the thickness of the coating layer is a crucial factor in different coated fabric applications [5,6]. Grecka et al. [5] investigated the abrasion and soaking properties of Cu- and Au-coated fabrics. They stated that thicker coating leads to greater bending stiffness. The authors concluded that too many layers can cause the cracking and peeling off of the coating layer. On the other hand, a greater number of coating layers ensures better filling of the concavities between weft and warp yarns, and, as a consequence, the fabric surface becomes smoother [5]. Mutlu et al. [6] showed that it is necessary to control the thickness of the nanoparticle coating layer while developing multifunctional textile composites. This ensures that both mechanical performance and fracture toughness are obtained. However, the authors investigated material coating using the dip solution-coating method [6].

The knife-coating technique was used to design a component of the artificial transverse muscles developed at Lodz University of Technology [7,8]. The artificial transverse muscle should be included as a third type of medical textile—a hybrid type. In this group are textiles used in external devices but that during diagnostics or treatment are temporarily introduced into the human body.

**Figure 1:**
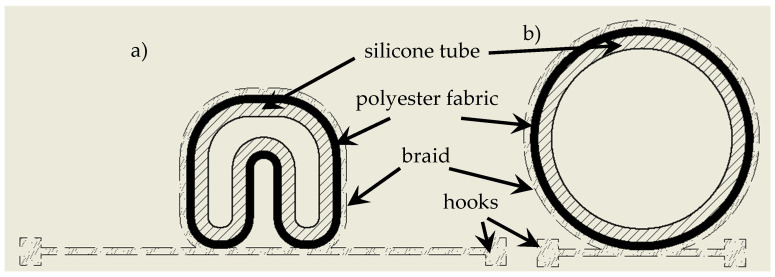
Structure of the artificial transverse muscle: (**a**) without compressed air supply; (**b**) supplied with compressed air.

The aim of the present work was to select the appropriate thickness of the silicone layer coating the PES woven fabric intended for use in artificial transverse muscles. The artificial transverse muscles are a component of a colonoscopy robot with a snake-like locomotion that could automatically retract itself into the patient’s body [9]. Due to its design and locomotion, its range would be unlimited and it would possess all the advantages of colonoscopes. Consequently, this type of robot increases the range of the colonoscope and allows for the diagnosis of areas of the intestines that have not been diagnosed [10]. To build such a robot, innovative artificial transverse muscles were developed [8]. They are constructed from a U-shaped silicone tube covered with polyester fabric coated with a thin layer of silicone, which protects the tube from over-inflation (Figure 1). A braid is then placed around the muscle’s circumference, with hooks placed at each end. The U-shaped tube is then closed with polyester fabric coated with a thin layer of silicone.

This type of the artificial muscle is unique and is used only by its inventors. Currently, these muscles are used only in medical applications.

In order to realize the artificial muscle, it was necessary to develop the textile components of it, i.e., the U-shape pipe coated by silicone. In the transverse muscle, the individual layers should have different physical properties. The polyester fabric coated with silicone layer which covers the muscle is primarily subjected to bending forces, while the front and side walls of the muscle, in addition to the bending load, must maintain their tightness under the pressure of the compressed air. Consequently, the serviceability of coated textile materials is closely related to the mechanical destruction of the coating layer. The flexing fatigue resistance is one of the tests applied in an assessment of durability of coating layer [11].

The assumption was that the artificial transverse muscle would be subjected to up to 10,000 cycles of fatigue bending during use. According to Figure 1 presenting the structure of the artificial transverse muscle, the coated PES fabric is a component of the developed artificial muscle. It creates a covering the U-shaped tube. Based on results of preliminary investigations, the polyester plain-woven fabric made of filament yarn was used as a covering for the artificial muscle. The assumption was that the PES fabric should be coated with silicone in order to ensure the tightness, smoothness, and evenness of the artificial muscle surface. Given that the U-shaped tube will be subjected to repeated bending while in use in the medical robot, an important task was to ensure resistance to fatigue bending.

## 2. Materials and Methods

In the U-shaped artificial muscle, a coated polyester woven fabric was applied as an external layer. The basic properties of the polyester substrate—the plain woven fabric—are presented in Table 1.

Two variants of coating by silicone were applied: 0.1 mm and 0.2 mm silicone layers. The experiment was limited to two variants because the thickness of the outer layer of the artificial muscle should be as small as possible while maintaining the expected functionality of the muscle. Preliminary investigations showed that it was not possible to achieve a coating layer thinner than 0.1 mm using this coating technique. While a thicker layer may increase the stiffness of the coated fabric, for the silicone layer, a key consideration was the effectiveness of the transvers artificial muscle, especially its movement. Due to this fact, based on preliminary investigations, the two aforementioned thickness variants of the silicone layer were applied in the present study.

**Figure 2:**
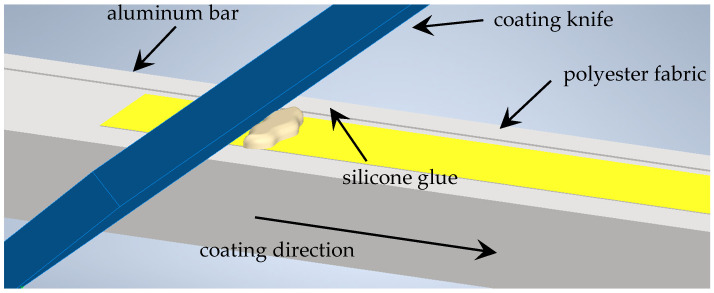
The scheme of coating procedure.

The process of coating was performed on an aluminum flat bar grounded in the middle at a depth of 0.1 mm and on the other side at a depth of 0.2 mm. Then, the strip of polyester fabric was placed in the grounded part of the bar. Following this, the silicone glue was placed at the beginning of the strip of fabric and the coating knife was pressed to the bar and pulled along it (Figure 2). Consequently, the silicone was pressed into the polyester fabric. The coating process was repeated a few times (3–5 times) on each polyester fabric strip to be sure that the coating process was performed correctly and that the silicone layer covered the entire fabric strip. This procedure was conducted for both thicknesses of the silicone layer: 0.1 and 0.2 mm.

In order to assess the smoothness of surface, the fabrics were tested with regard to their surface roughness. Measurement was performed by means of the 3D contactless method using the optical technique applied in the MicroSpy^®^ Profile profilometer, FRT, the art of metrology, Bergisch Gladbach, Germany [15,16]. The profilometer provides precise measurement of a surface geometry. It is equipped with a patented chromatic white-light (CWL) sensor which utilizes the chromatic aberration of optical lenses [11]. Measurement was performed for the uncoated and coated fabrics. For each variant, five specimens were measured. Additionally, the surface geometry was assessed for the fabrics after the fatigue bending process.

First, the images of the fabric surface were prepared by means of the profilometer. Next, the pictures were processed in the specialist Mark II software, version 3.11, by the FRT and the art of metrology, Bergisch Gladbach, Germany [17,18].

**Figure 3:**
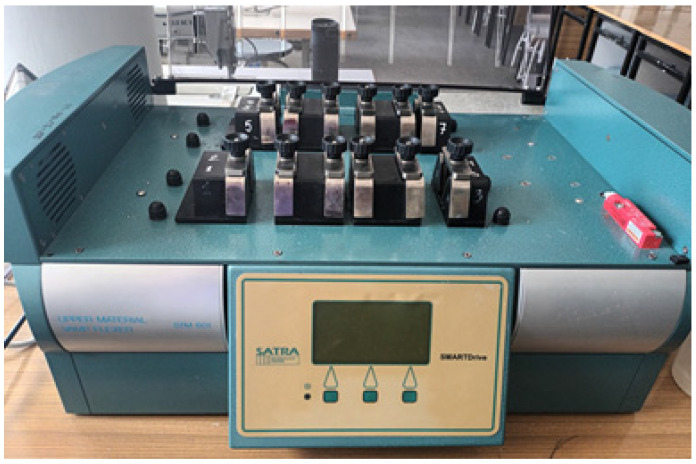
The STM 601/12 instrument for fatigue bending test.

Determination of the resistance of coated fabrics to fatigue bending loads was performed by means of the STM 601/12 device (Figure 3), manufactured by SATRA (Northamptonshire, UK) [19].

**Figure 4:**
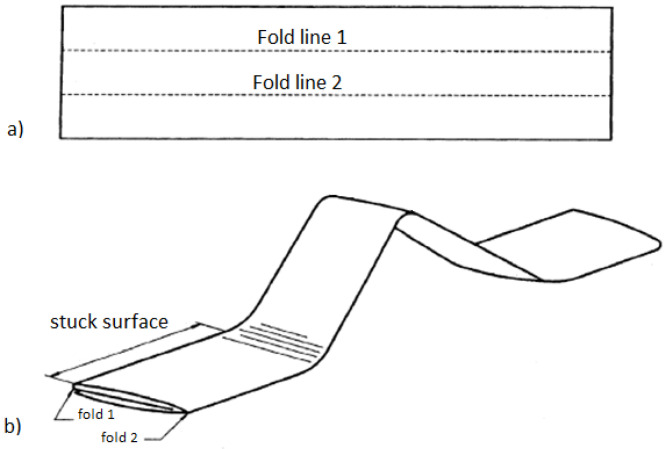
The scheme of sample preparation for measurement by the STM 601/12 device: (**a**) sample of dimensions: 37.5 mm by 125 mm; (**b**) folded sample.

This instrument assesses the tendency of textile materials to crack or break as a result of flexing in wear. The method of mounting samples in the workstations was performed in accordance with the PN-EN-ISO 7854:2002 standard [20]. The De Mattia method was used, which involves preparing samples with dimensions of 37.5 ± 1 mm by 125 ± 1 mm (Figure 4a). The prepared samples were folded twice, as shown in Figure 4b, with the coating facing outward.

**Figure 5:**
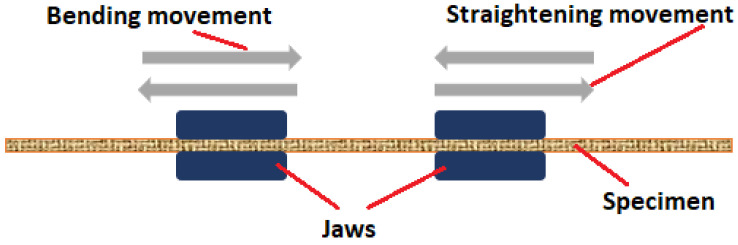
The scheme of specimen clamping in the STM 601/12: device jaws and bending movement of jaws.

The slightly tensioned sample was then clamped in the device’s grips (Figure 5). The jaws then move cyclically in a horizontal direction, approaching and moving apart, which causes the test sample to cyclically bend and straighten.

Sample bending occurs as the clamps of the device approach and recede from each other. Thus, the length between jaws is changeable and changes at the same speed during the test. The maximum (initial) distance between the clamps is 30 mm and the minimum is 5 mm. Due to this fact, it is impossible to establish an investigated area equal to the distance between jaws. The maximum bending was usually observed in the middle zone between the clamps, but not exactly in the center. To assess the surface geometry, the central part of the sample fragment between the clamps was used. Figure 4 only schematically shows what the sample prepared for bending looks like and an exact representation of the shape of the sample during the test is not shown. In reality, the samples buckled either upwards or downwards (randomly) as a result of bending forces.

After stopping the device after a specified number of strokes, a check for cracks on the material surface was performed by organoleptic observation and by analysis of microscopic images of samples. For microscopic observations, the MMT, SZX 10 stereomicroscope, Evident Europe GmbH, Hamburg Germany was applied. An assessment of the specimens’ surfaces was performed after 4000, 10,000, and 20,000 cycles of bending. Initially, 4000 flexing cycles were assumed as a preliminary value. If, after 4000 flexing cycles, changes were observed that would disqualify the coated fabric for use in artificial muscles, the study would not be continued. The next threshold was 10,000 flexing cycles, the number of cycles that, according to the design assumptions, would occur during the use of the artificial muscles. The next threshold was 20,000 flexing cycles, which is twice the number of cycles assumed during muscle use. This number was adopted to ensure an adequate safety margin.

The microscopic observations of the coated fabric surface were also performed for samples before the cyclic bending tests.

It should be mentioned here that during use, the artificial muscles will be alternately filled with air and deflated to create a movement effect within the intestine. The artificial transverse muscle will be surrounded by bodily fluids on the outside. No additional external mechanical stresses will occur during use, so testing was conducted only within the scope presented. It should also be noted that the coated polyester fabric will only serve as a cover for the U-shaped tube. All stresses during use will be transferred through the entire material system in the artificial transverse muscle, as shown in Figure 1. Therefore, the scope of testing for the coated polyester fabric being a muscle component was determined as presented in this article.

All experimental data were analyzed using statistical tools: one- and multifactor analysis of variance (ANOVA). The parameters characterizing the surface geometry were taken as dependent variables, whereas the thickness of the coating layer and number of bending cycles were taken as the main factors. The statistical significance of the stated relationships was assessed at a 0.05 significance level.

## 3. Results

**Figure 6:**
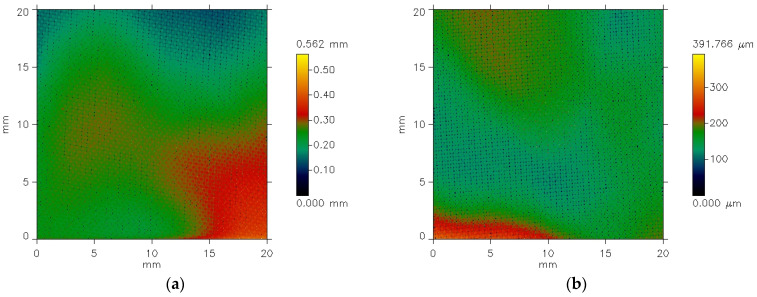
The exemplary images of the PES fabric specimens: (**a**) specimen 3; (**b**) specimen 4.

Figure 6 presents the examples of pictures of the polyester woven fabric before coating. Figure 6 presents the pictures of fabric specimens’ surface being measured. The pictures are made by the profilometer. The established area of the measured surface is 20 mm (x-axis) by 20 mm (y-axis). The color of the picture indicates the height of particular points in the measured area according to the color scale (scale bar) presented on the right side of the pictures. The scale bar illustrates the height of individual points on the measured area. It is clearly seen that the height of the points on the measured area is diversified from 0 mm to more than 0.56 mm for specimen 3 (Figure 6a) and from 0 mm to 0.39 mm for specimen 4 (Figure 6b). This is due to the fact that the fabrics do not adhere precisely to the surface of the device table. In order analyze the surface of the investigated fabric specimens, it was necessary to process the images from the profilometer in the specialist Mark III software, version 3.11 [17,18]. Two steps were necessary:

- First, the elimination of error resulting inaccurate specimen placement on the instrument table; cut-off filter λc = 0.8 mm.
- Second, the elimination of noise; noise filter λs = 0.1 mm.

**Figure 7:**
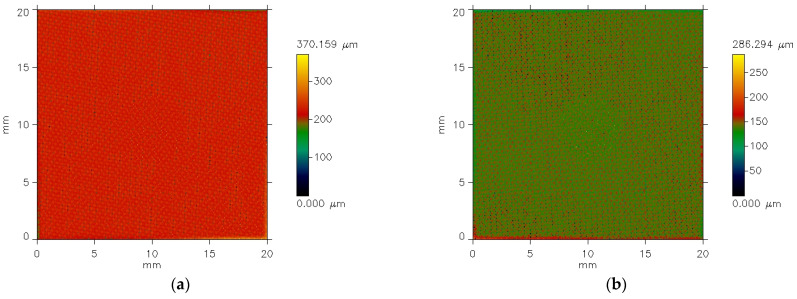
The exemplary images of the PES fabric specimens after elimination of waviness and noise: (**a**) specimen 3; (**b**) specimen 4.

For this purpose, the appropriate filters were applied: noise filter—λn = 0.1 mm and the cut-off wavelength filter λc = 0.8 mm. The results of the filtering are presented in Figure 7.

Next, the parameters characterizing the surface geometry were calculated in accordance with the ISO standard [20]. The same procedure was applied for the measurement of the coated fabrics. According to the standard [21], several parameters can be calculated. Next, they were analyzed statistically in order to assess the statistical significance of their changes due to the coating process. Table 2 presents the values of the parameters, which changed significantly as a result of the coating process. These are the following parameters:

- Amplitude average parameters: Ra, Rq, Rku.
- Amplitude parameter: Rv.
- Parameters of the surface having stratified functional properties: Rk and Rvk.

The Ra parameter is described in [21] as an arithmetic mean of the absolute of the ordinate values within an analyzed area. This parameter represents a difference between the height of each point of the measured surface and the arithmetical mean height of the surface. On the other hand, the Rq parameter is defined as root mean square value of the ordinate values within a tested area. The Rq parameter relates to the standard deviation of height of all points on the measured surface. Kurtosis (Rku) relates to geometry of peaks and valleys. This parameter is used for the evaluation of the degree of contact between two objects. This should be discussed as follows:

- When Rku is equal to 3, a height distribution is considered as normal;
- In the case where Rku is greater than 3, a height distribution is sharp;
- A Rku value smaller than that means that the height distribution is even.

The amplitude parameter Rv represents the maximum depth of valleys on the measured surface.

Both functional parameters, Rk and Rvk, are derived from the material ration curve (also called the Abbott–Firestone curve). The curve presents the percentage of material that is visible while slicing through a surface at various depths. The assumption is that the surface of different solid objects consists of three components: core (central), peaks, and valleys. Consequently, three parameters are used for characterization of surface heights: the core roughness depth Rk (representing the central part of the Abbott–Firestone curve), reduced peak height Rpk, and reduced valley depth Rvk [22].

The results of surface geometry measurements of both raw and coated polyester woven fabric are presented in Table 2. Standard deviation of results are shown in parentheses.

**Figure 8:**
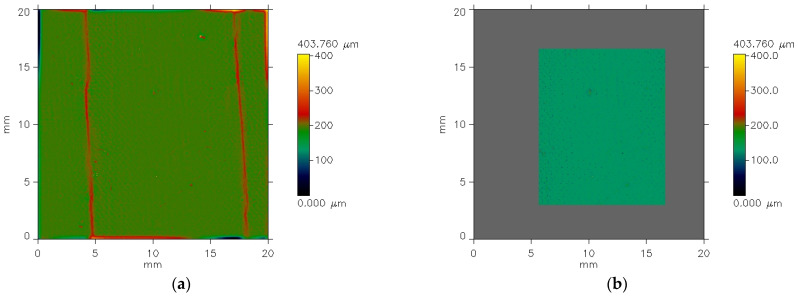
The exemplary filtered images of the specimens after fatigue bending test: (**a**) whole area of sample image; (**b**) isolated are without the sharp edges.

In the specimens, after the fatigue bending by means of the SATRA, the sharp edges occurred in places where the samples were folded (Figure 8—red lines). The curvatures that appear in the sample after fatigue bending are a result of the sample being clamped in the device, not the bending itself. Therefore, we decided to test the sample area which was not subjected to compression by the device clamps. Furthermore, we analyzed the surface roughness, which is a set of surface irregularities that may or may not repeat periodically and are characterized by the fact that the ratio of the average width of the elements to their average height is less than 40 [23,24]. Roughness is one of the three components of a surface’s geometric structure. Alongside roughness are waviness and shape errors [25]. Waviness is defined as a set of periodically repeating elements, characterized by the fact that the ratio of the average width of the profile elements to their height is in the range of 40 to 1000 [23,24]. Shape concerns an overall, large-scale geometry of the surface which ignores the shorter variations in roughness and waviness. In the case of textiles, usually, shape is not analyzed because fabrics are flexible materials considered as flat. Additionally, a fabric’s shape can change even under the influence of the weight of these materials themselves. During the analysis, long-term changes in waviness and shape variability were eliminated using appropriate options available in the software—cut-off filter. In the present study, both components, waviness and shape errors, of the coated fabric surface topography are not inherent properties of the tested materials. They result from the imprecise adjacency of the sample to the profilometer’s measuring table, and in the case of samples after cyclic bending, they are also a result of jamming in the device clamps. Applying an appropriate filter allows us to eliminate the effects of waviness and shape errors, i.e., sample curvature, and assess pure roughness. This is a standard procedure in roughness analysis. Next, it was necessary to isolate the area without the sharp edges. For the isolation of this area, the Modification function available in the Marek II software was used, with the option of Masking. For analysis of each specimen after fatigue bending, a uniformly long area (14 mm in the y-axis direction), located centrally between the sample clamping points, was isolated (Figure 8b).

Table 3 presents the results of the surface geometry measurement of coated fabrics after 4000, 10,000, and 20,000 cycles of bending.

It is clearly seen that the parameters characterizing the surface geometry of fabric have been changed due to fatigue bending, depending on the number of bending cycles. Some of them increased in comparison to the parameters before fatigue bending, while other ones decreased. A more detailed analysis of the results is presented in the next chapter.

## 4. Discussion

The results of fabric surface geometry testing confirmed that the coating process influenced the surface characteristic of the polyester fabric being investigated. The influence of coating on the surface geometry of the investigated polyester woven fabrics was assessed by means of statistical analysis using one-way ANOVA. While analyzing, the surface geometry parameters were taken as the dependent variables, whereas the thickness of the coating layer was taken as the main factor. The statistical analysis confirmed that the values of such parameters as Ra, Rq, Rk, and Rvk decreased significantly after the coating process. Additionally, the values of both parameters for fabric with 0.2 mm coating layer were significantly smaller than that for the fabric with 0.1 mm coating layer (Table 2), except for the Rvk parameter. The coating process caused a shallowing of the valleys on the fabric surface by filling the valleys with silicone. This then resulted in a reduction in the Ra and Rq values. The value of kurtosis (Rku) increased significantly due to the coating process. However, in all cases, the value of kurtosis is significantly higher than 3. This means that the distribution of the points’ height (z-value) on the tested area is sharp for the fabric both before and after coating. Coating caused significant sharpening the height distribution. It was also stated that kurtosis for the fabric with a 0.2 mm coating layer was greater than that for the fabric with a 0.1 mm coating layer.

**Figure 9:**
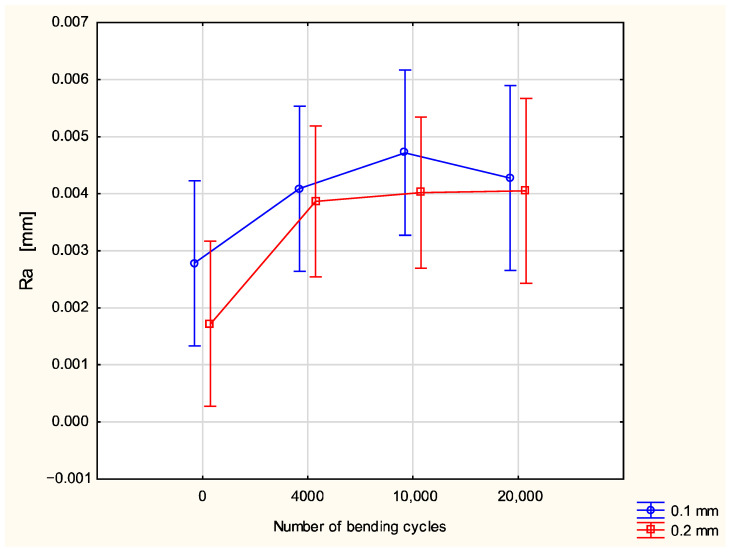
The Ra parameter in function of number of bending cycles and thickness of coating layer.

The results for coated fabric after fatigue bending showed an influence of both the number of bending cycles and a thickness of coating layer on parameters characterizing the fabric’s surface geometry. In order to assess the statistical significance of changes in the fabrics’ surface geometry quality due to the fatigue bending, a statistical analysis was performed using multi-factor ANOVA. The values of surface geometry parameters were taken as the dependent variables, whereas the number of bending cycles and the thickness of coating layer were taken as the main factors. Results for the raw polyester fabric and fabric after coating confirmed that the coating process influenced the surface geometry of investigated polyester fabric. The value of the Ra parameter increased as a result of fatigue bending (Figure 9). Due to a great variation in the results in the frame of the given variants, the influence of both main factors—the number of bending cycles and the thickness of the coating layer—was assessed as statistically insignificant. However, it is visible that the increase in the Ra value due to the fatigue bending process for the fabric with a 0.2 mm coating layer is smaller than that for the fabric with 0.1 mm coating layer.

**Figure 10:**
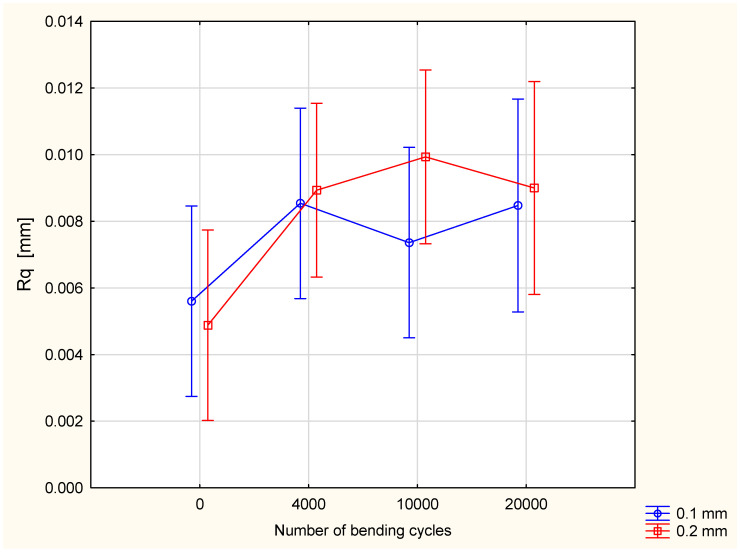
The Rq parameter in function of number of bending cycles and thickness of coating layer.

Statistical analysis confirmed a statistically significant influence of the number of bending cycles on the Rq value. With the increase in the number of bending cycles, the value of the Rq parameter also increased (Figure 10). The influence of the silicone layer thickness on the Rq value was assessed as statistically insignificant, with a 0.05 significance level. The interaction between the main factors is also statistically insignificant.

**Figure 11:**
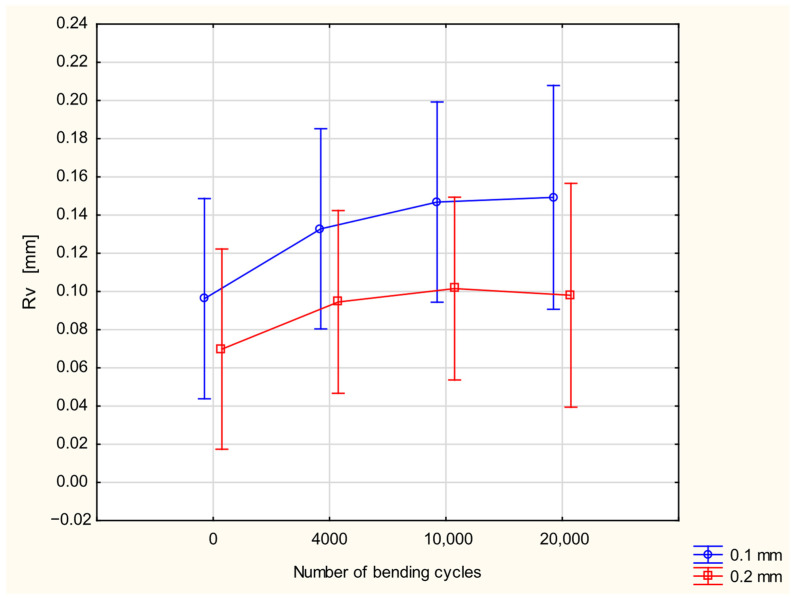
The Rv parameter in function of number of bending cycles and thickness of coating layer.

The opposite relationships were observed for the Rv parameter, the maximum valley depth of a surface within the sampling area. The Rv values for fabric coated with 0.2 mm silicone layer are significantly smaller than that for the fabric with a 0.1 mm silicone layer (Figure 11). However, attention should be paid here to the fact that the maximum valley depth in fabric coated by the 0.2 mm silicone layer before fatigue bending was also significantly smaller than that for the fabric coated by the 0.1 mm silicone layer. The Rv value increases with the increase in the number of bending cycles; however, the influence of the number of bending cycles on the Rv value was assessed as statistically insignificant.

**Figure 12:**
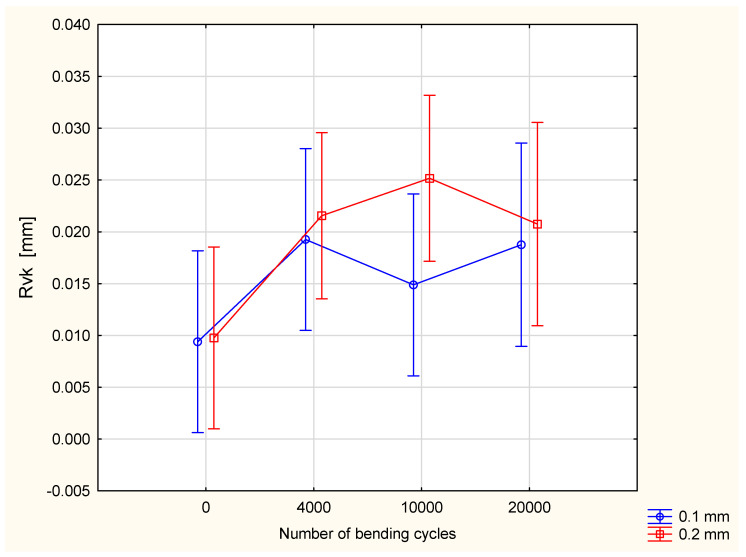
The Rvk parameter in function of number of bending cycles and thickness of coating layer.

In the case of the Rvk parameter—reduced valley depth—the relationship is similar to that observed for the Rq parameter. The number of bending cycles influences the Rvk value in a statistically significant way, at a 0.05 significance level. With the increase in the number of bending cycles, the value of the Rvk parameter also increases (Figure 12). The influence of silicone layer thickness on the Rq value as well as an interaction between the two were assessed as statistically insignificant.

**Figure 13:**
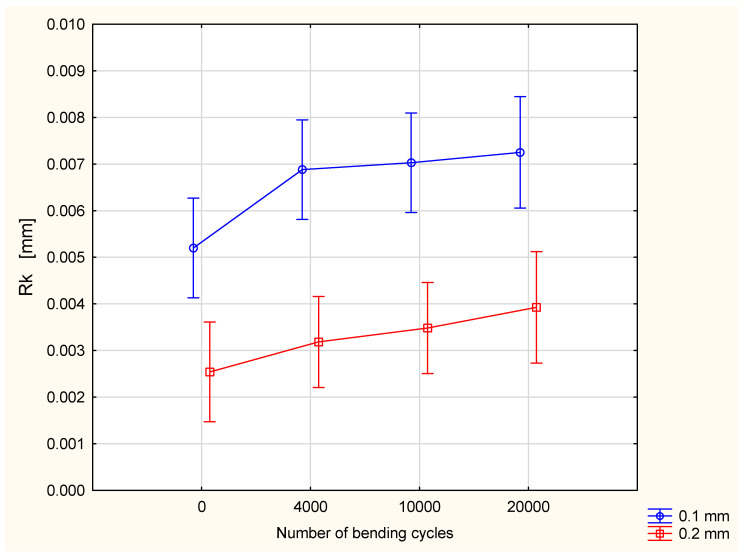
The Rk parameter in function of number of bending cycles and thickness of coating layer.

Very clear tendencies were observed for the Rk parameter—core roughness depth (Figure 13). The Rk value increases with the increase in the bending cycle number. Fabric with a 0.2 mm silicone layer is characterized by a significantly smaller value of the Rk parameter in comparison with the Rk value for fabric with a 0.1 mm silicone layer. Both the number of bending cycles and the thickness of the coating layer influence the Rk value in a statistically significant way.

Analysis of the surface geometry of the polyester fabric before and after coating showed that coating resulted in a smoothing of the fabric surface, mainly as a result of filling the pore cavities of the fabric surface with silicone. This is confirmed by the values of parameters characterizing the surface valley depth such as Rv and Rk. Additionally, it was shown that coating with a 0.2 mm thick silicone layer resulted in a smoother surface than coating with a 0.1 mm thick silicone layer.

**Figure 14:**
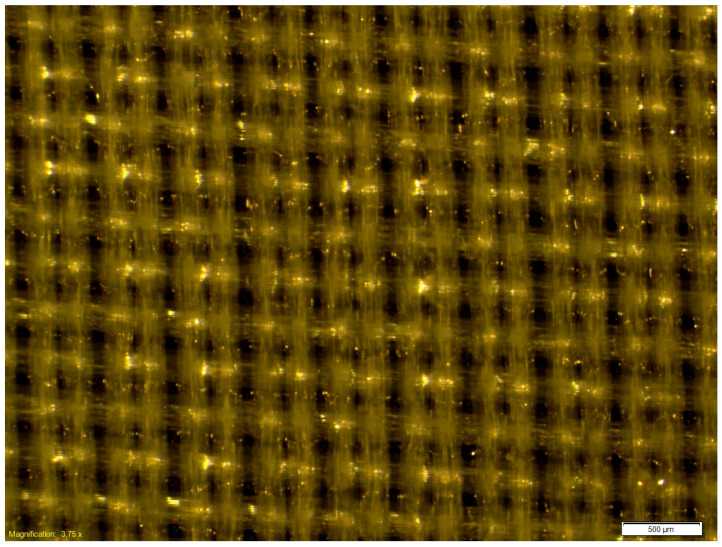
The exemplary microscopic pictures of the polyester woven fabric coated with 0.1 mm silicone layer before fatigue bending.

**Figure 15:**
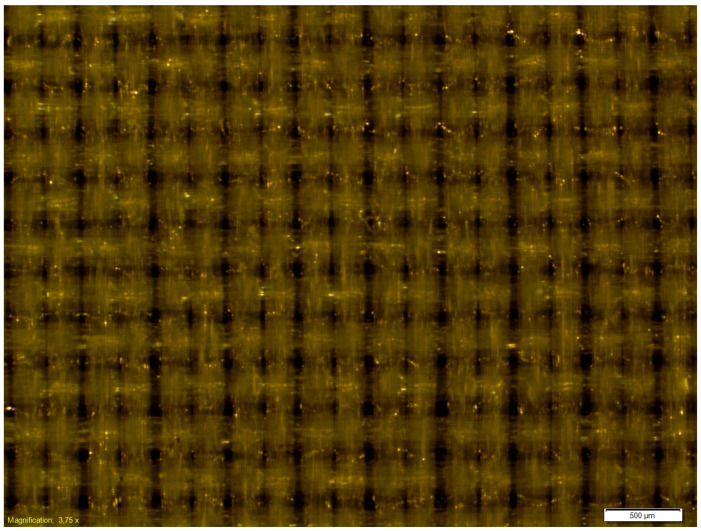
The exemplary microscopic pictures of the polyester woven fabric coated with 0.2 mm silicone layer before the fatigue bending.

**Figure 16:**
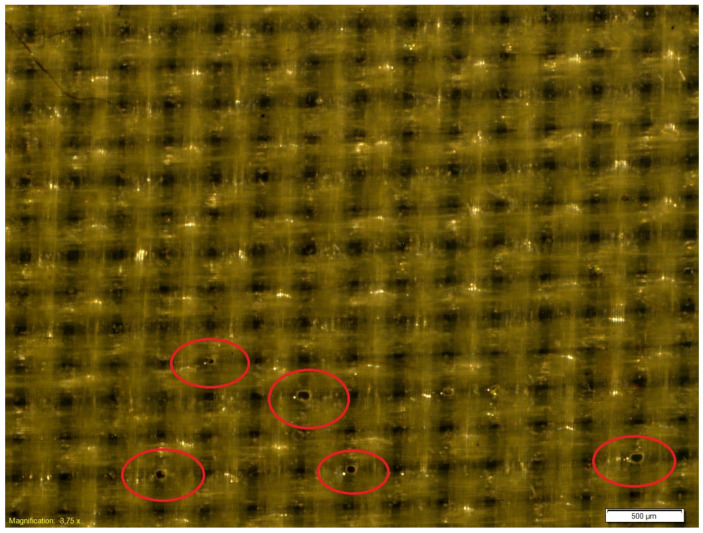
The exemplary microscopic pictures of the polyester woven fabric coated with 0.1 mm silicone layer after 10,000 bending cycles. The red circles show exemplary damaged areas—holes in silicon.

**Figure 17:**
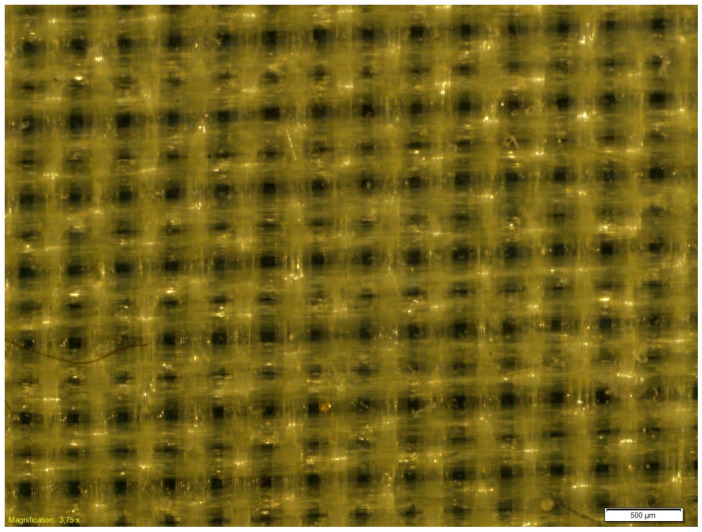
The exemplary microscopic pictures of the polyester woven fabric coated with 0.2 mm silicone layer after 10,000 bending cycles.

The microscopic observations of the coated samples were performed before and after cycling bending. Exemplary microscopic pictures of coated fabrics before the fatigue bending are presented in Figure 14 and Figure 15. Any local damage was observed before the cyclic bending. The microscopic observations of coated samples after the fatigue bending process confirmed the significant advantage of the fabric coated with a 0.2 mm silicone layer over fabric coated with a 0.1 mm silicone layer. After 10,000 bending cycles, local damage to the silicone layer was visible in the fabric coated with a thinner silicone layer (Figure 16). Such damage was not observed in the fabric coated with a 0.2 mm thick layer of silicone (Figure 17).

The presented study investigated differences in surface roughness of fabrics coated with a silicone layer with a thickness of 0.1 mm or 0.2 mm. Roughness measurements were conducted after subjecting the fabrics to varying numbers of bending cycles. The results indicate that increased bending leads to higher surface roughness of the coated layer. However, no significant wear or degradation of the silicone layer was observed organoleptically as well as under a microscope. Investigations confirmed the assumption that a 0.2 mm thick silicone coating is appropriate for the intended use. The tests used a total of 20,000 bending cycles, while the expected number of bending cycles during usage in artificial muscles is half that, i.e., 10,000 cycles. These findings support the feasibility of using this technology in the construction of transverse artificial muscles, as the silicone-coated fabric demonstrates sufficient durability. It can therefore be concluded that the muscles should withstand the intended 10,000 operating cycles.

Microscopic analysis of the 0.1 mm thick silicone-coated fabric revealed minor losses in the silicone layer, which may compromise its sealing properties. In contrast, the 0.2 mm thick fabric showed no such defects, indicating its structural integrity both before and after fatigue testing. Based on these results, the 0.1 mm thick fabric may be suitable for use as a reinforcing layer over silicone tubing in transverse muscle construction, where the tube ensures airtightness and the fabric provides mechanical support against excessive deformation. Meanwhile, the 0.2 mm thick fabric is recommended for use in the front and rear walls of the muscle, as it maintains sealing performance throughout the fatigue cycle.

## 5. Conclusions

The investigations performed showed that coating the polyester plain woven fabric with a silicone layer of 0.2 mm ensures greater resistance to fatigue bending that coating it with layer of 0.1 mm. The microscopic pictures of fabrics after fatigue bending showed some holes in the silicone with a 0.1 mm silicone layer. The holes were created after fatigue tests. Such holes were not observed in the pictures fabric coated with a 0.2 mm silicone layer. Additionally, it was stated that fabric coated with a 0.2 mm silicone layer was characterized by a smoother and more even surface than the fabric with a 0.1 mm silicone layer. This is confirmed by the values of such parameters as Ra and Rq, which are lower for the fabric coated with a 0.2 mm silicone layer than for fabric coated with a 0.1 mm silicone layer.

## Figures and Tables

**Table 1:** The basic properties of the polyester plain woven fabric.

Parameter	Unit	Value	Method
Mass per square meter	g/m^2^	53	PN-ISO 3801:1993 [12]
Warp density	threads/cm	33	PN-EN 1049-2:2000 [13]
Weft density	threads/cm	33	PN-EN 1049-2:2000 [13]
Thickness	mm	0.08	PN-EN ISO 5084:1999 [14]

**Table 2:** The results from the profilometer for uncoated and coated fabric.

Variant	Ra (SD) mm	Rq (SD) mm	Rk (SD) mm	Rvk (SD) mm	Rku (SD) mm
Raw fabric	0.0089 (0.0009)	0.0148 (0.0016)	0.0220 (0.0012)	0.0338 (0.0041)	23.67 (11.92)
Coated 0.1 mm	0.0035 (0.0015)	0.082 (0.0044)	0.0066 (0.0021)	0.0142 (0.0063)	84.28 (34.31)
Coated 0.2 mm	0.0023 (0.0011)	0.0076 (0.0036)	0.0029 (0.0013)	0.0152 (0.0083)	89.79 (38.94)

**Table 3:** The results from the profilometer-coated fabrics after fatigue bending.

Variant	Ra (SD) mm	Rq (SD) mm	Rk (SD) mm	Rvk (SD) mm	Rku (SD) mm
After 4000 bending cycles					
Coated 0.1 mm	0.0041 (0.0006)	0.0085 (0.0018)	0.0069 (0.0009)	00.0193 (0.0019)	43.52 (21.14)
Coated 0.2 mm	0.0039 (0.0022)	0.0089 (0.0047)	0.0031 (0.0010)	0.0216 (0.0153)	45.41 (18.61)
After 10,000 bending cycles					
Coated 0.1 mm	0.0047 (0.0025)	0.0074 (0.0021)	0.0070 (0.0014)	0.0145 (0.0059)	87.80 (65.28)
Coated 0.2 mm	0.0040 (0.0018)	0.099 (0.0035)	0.0035 (0.0010)	0.0252 (0.0126)	134.00 (245.73)
After 20,000 bending cycles					
Coated 0.1 mm	0.0043 (0.0010)	0.0085 (0.0027)	0.072 (0.0010)	0.0188 (0.0094)	32.18 (29.89)
Coated 0.2 mm	0.0040 (0.0011)	0.0090 (0.0024)	0.0039 (0.0012)	0.021 (0.0097)	97.41 (74.53)

## Data Availability

The original contributions presented in this study are included in the article. Further inquiries can be directed to the corresponding author.
